# Long-Term Redistribution of Peripheral Lymphocyte Subpopulations after Switching from Calcineurin to mTOR Inhibitors in Kidney Transplant Recipients

**DOI:** 10.3390/jcm9041088

**Published:** 2020-04-11

**Authors:** Laura Llinàs-Mallol, Dolores Redondo-Pachón, Dàlia Raïch-Regué, María José Pérez-Sáez, José Yélamos, Xavier Duran, Anna Faura, Miguel López-Botet, Julio Pascual, Marta Crespo

**Affiliations:** 1 Department of Nephrology; Hospital del Mar, 08003 Barcelona, Spain; 2 Nephropathies Research Group, Hospital del Mar Medical Research Institute (IMIM), 08003 Barcelona, Spain; 3 Department of Immunology, Hospital del Mar, 08003 Barcelona, Spain; 4 Methodology & Biostatistics Support Unit, IMIM, 08003 Barcelona, Spain; 5 Immunity and Infection Research Group, IMIM, 08003 Barcelona, Spain; 6 University Pompeu Fabra, 08003 Barcelona, Spain

**Keywords:** donor-specific antibody, immunophenotype, kidney transplantation, mTOR inhibitors, T regulatory cells, transitional B cells, NK cells

## Abstract

Classical immunosuppression based on steroids, calcineurin inhibitors, and mycophenolate results in several unwanted effects and unsatisfactory long-term outcomes in kidney transplantation (KT). New immunosuppressors search for fewer adverse events and increased graft survival but may have a distinct impact on graft function and immunological biomarkers according to their mechanism of action. This prospective study evaluates the immunological effect of tacrolimus to serine/threonine protein kinase mechanistic target of rapamycin inhibitors (mTORi) conversion in 29 KT recipients compared with 16 controls maintained on tacrolimus. We evaluated renal function, human leukocyte antigen (HLA) antibodies and peripheral blood lymphocyte subsets at inclusion and at 3, 12, and 24 months later. Twenty immunophenotyped healthy subjects served as reference. Renal function remained stable in both groups with no significant change in proteinuria. Two patients in the mTORi group developed HLA donor-specific antibodies and none in the control group (7% vs. 0%, *p* = 0.53). Both groups showed a progressive increase in regulatory T cells, more prominent in patients converted to mTORi within the first 18 months post-KT (*p* < 0.001). All patients showed a decrease in naïve B cells (*p* < 0.001), excepting those converted to mTORi without receiving steroids (*p* = 0.31). Transitional B cells significantly decreased in mTORi patients (*p* < 0.001), independently of concomitant steroid treatment. Finally, CD56^bright^ and CD94/NK group 2 member A receptor positive (NKG2A^+^) Natural Killer (NK) cell subsets increased in mTORi- compared to tacrolimus-treated patients (both *p* < 0.001). Patients switched to mTORi displayed a significant redistribution of peripheral blood lymphocyte subpopulations proposed to be associated with graft outcomes. The administration of steroids modified some of these changes.

## 1. Introduction

Kidney transplantation (KT) is the treatment of choice for end-stage renal disease, given the improvement in life expectancy and quality comparing with long-term dialysis [1,2,3,4]. Despite the good results in short-term graft survival rates, half-life of renal allografts is around 10 years under immunosuppression treatment mainly based on steroids, calcineurin inhibitors (CNI), and antiproliferative agents [5,6,7]. Regardless of the use of this immunosuppressive strategy, development of antibody-mediated rejection (ABMR), a form of rejection associated with donor-specific antibodies (DSA), is a major cause of graft loss [8,9,10,11,12]. Death accounts for around half of graft-losses, mainly due to cardiovascular disease, cancer, or infections [13,14]. These results reflect an unmet need of new immunosuppressive therapeutic regimens more efficient for improving long-term survival rates.

The introduction of the serine/threonine protein kinase mechanistic target of rapamycin (mTOR) inhibitors in transplantation pursued the challenge of reducing nephrotoxicity related to the use of CNI [15], so as to increase the life of the graft and to decrease the risk of cancer [16] or infections [17]. The intracellular mTOR complex has an important role in the modulation of both innate and adaptive immune responses [18,19]. Inhibition of mTOR displays systemic effects in cell proliferation and has different immunomodulatory effects on antigen-presenting cells, T cells, B cells, Natural Killer (NK) cells, neutrophils, and mast cells [18,19]. It has been reported that mTOR inhibitors (mTORi) stimulate the proliferation of T regulatory (Treg) cells, both in vitro [20,21] and in vivo [22,23,24,25,26]. Currently, there is controversial data regarding the effect of mTORi conversion in other peripheral blood T cell subsets [20,27,28,29]. Moreover, scarce and controversial data are available regarding peripheral blood B cells and NK cells and the use of mTORi [27,30,31,32,33,34].

The development of de novo DSA (dnDSA) has been recognized as a risk factor for graft loss, associated with the development of ABMR [35]. Contradictory data exist regarding the influence of mTORi on the development of dnDSA [36,37,38,39,40,41]. These controversial results may be due, among other variables, to differences in time post-transplant and on treatment; in dosage and type of mTORi, as well as to concomitant immunosuppressive drugs employed.

Here, we aimed to perform a case-control study of the influence of mTORi conversion on graft function, development of dnDSA, and peripheral blood lymphocytes (PBL) with an extensive prospective follow-up of 2 years.

## 2. Experimental Section

### 2.1. Study Design and Population

We designed a prospective observational study to evaluate the potential impact of mTORi treatment on the development of human leukocyte antigen (HLA) antibodies in kidney transplant recipients (KTR) and the distribution of PBL subsets. From April 2011 to December 2015, we recruited KTR who switched from tacrolimus to either everolimus or rapamycin (11 within clinical trials and 18 for clinical reasons, 13 of them due to previous malignancies) and a contemporaneous group of patients maintained on tacrolimus, and who were followed until February 2018. At inclusion, all 45 patients but one—on cyclosporine—received immunosuppression with tacrolimus (mean trough blood level 7.9 ng/mL) with or without concomitant mycophenolate acid (*n* = 39, mean dose 598 mg/day) and prednisone (*n* = 35, 5 mg/day). Clinical evaluation (serum creatinine, estimated glomerular filtration rate (eGFR) by Modification of Diet in Renal Disease Study equation (MDRD-4) and proteinuria measured as protein/creatinine in mg/g urine), HLA antibody analysis, and fresh blood immunophenotyping were performed before and 3, 12, and 24 months after mTORi conversion or inclusion. In addition, PBL subsets of 20 healthy subjects (HS) were also analyzed. The study was approved by the Parc de Salut Mar Ethical Research Board (2011/4385/I), and all patients gave written informed consents. Clinical and research activities being reported herein are consistent with the Principles of the Declaration of Istanbul and the Declaration of Helsinki. No organs were procured from prisoners.

### 2.2. Determination of HLA Antibodies

Serum samples were collected and stored at −80 °C until analysis. Screening for anti-HLA antibodies was performed with Luminex Lifecodes LifeScreen Deluxe assay (Gen-probe^®^, Stamford, CT, USA), and anti-HLA alloantibody identification was performed using Lifecodes LSA Class-I (93 beads) and/or Class-II (84 beads) assays (Gen-probe^®^, Stamford, CT, USA), as previously described [42]. Donor HLA antibody specificity was ascribed following the results of single antigen assays, considering donor HLA typing or linkage disequilibrium for HLA-C or HLA-DQ antigens when typing was not fully available. A reaction with mean immunofluorescence intensity over 1000 was considered positive.

### 2.3. Immunophenotyping Analysis

Immunophenotyping was performed by flow cytometry on fresh peripheral blood samples, obtained by venous puncture in ethylenediamine tetraacetic acid (EDTA) tubes. Samples were pretreated with saturating concentrations of human-aggregated immunoglobulins to block antibody constant region heavy chain γ receptor (FcγR) and then labelled with different antibody combinations to define T, B and NK-cell subsets in separated tubs as described in Reference [43] (Table S1 and Figure S1). Samples were acquired by a FACS Canto II cytometer, and data were analyzed by FACS Diva v.7 and FlowJo v.10 softwares (BD Biosciences™, Franklin Lakes, NJ, USA), as described [43]. CD3^+^ T lymphocytes including CD4^+^ and CD8^+^ subsets were identified. B lymphocytes were characterized as CD19^+^ cells, and subpopulations were analyzed considering IgD and either CD27 or CD38 expression [44]. For this study, CD3^−^CD56^+^ NK cell subsets were defined according to CD56 fluorescence intensity (CD56^bright^ and CD56^dim^) and to CD94/NK group 2 member A receptor (NKG2A) and CD94/NK group 2 member C receptor (NKG2C) expression (Figure S1). Absolute cell numbers were calculated from parallel blood counts. Validation of the transitional B cell immunophenotype was performed as previously designated [43] (Figure S2).

### 2.4. Statistical Analysis

We performed an on-treatment analysis considering data of patients at each study point if they stayed on the intended treatment. Comparisons between normally distributed variables were carried out by using Student’s t-test, and nonparametric variables were analyzed with U Mann–Whitney test. Normal distribution of continuous variables was tested with Kolgorov–Smirnoff and Shapiro–Wilk tests. Chi-squared or Fisher’s exact tests were used for dichotomous variables. Generalized Estimating Equations (GEE) population-averaged model was used for analyzing changes in PBL subpopulations, including an interaction term in order to check differences between study groups. Two *p*-values were obtained, one for each study group and PBL subpopulation evolution (therefore representing the comparison between baseline and 3-, 12-, and 24-month data) and another one evaluating the differences between the two groups of study in the evolution of each PBL subpopulation. Analysis of T regulatory cells was also adjusted by time after KT, sex, age, and delayed graft function. A *p*-value < 0.05 was considered statistically significant. Statistical analysis was performed using SPSS^®^ v.22.0 (IBM Corp, New York, NY, USA) and Stata^®^ v.15 (STATA Corp, College Station, TX, USA).

### 2.5. Data Availability

The datasets generated and analyzed during the current study are not publicly available but are available from the corresponding author on reasonable request.

## 3. Results

### 3.1. Study Population and Clinical Follow-Up

Forty-five patients with stable renal function were included in the study: 29 switched from CNI to mTORi (25 everolimus and 4 rapamycin, mTORi group), and 16 maintained treatment with tacrolimus, steroids, and mycophenolic acid (Tacrolimus group). Twenty-two converted patients and all 16 tacrolimus patients finalized the 24-month study period on treatment. Seven recipients did not complete 24 months on mTORi: six reintroduced tacrolimus between 12 and 24 months due to surgery (three cases), ABMR (two cases), and proteinuria; and one patient died 19 months after conversion for metastatic prostate carcinoma. Baseline and 3- and 12-month data of these patients were included in the analysis. No graft loss was observed during the study period. Main characteristics of both groups are included in Table 1. All tacrolimus and 19 of 29 mTORi patients received prednisone during the study. At 24 months, tacrolimus trough blood levels remained stable in the tacrolimus group (6.5 ng/mL), mTORi trough blood levels were 6.8 ng/mL in the mTORi group, and MPA dose was similar in both groups (566 mg in the tacrolimus group and 725 mg in the mTORi group). Clinical follow-up showed stable renal function and a non-significant increase in proteinuria in the mTORi group (Table 1).

### 3.2. Conversion from Tacrolimus to mTOR Inhibitor was not Associated with a Significant Development of de novo Donor Specific Antibodies

During the study, 7 patients showed de novo HLA antibodies: five in the mTORi group, two of them HLA DSA (one class I and one class II, 6.9%) and three HLA no DSA (10%), and two patients in the tacrolimus group had de novo HLA class II no DSA (13%). Rates of HLA no DSA (mTORi: 10% vs. tacrolimus: 13%, *p* = 1.00) and anti-HLA dnDSA (mTORi: 7% vs. tacrolimus: 0% *p* = 0.53) were statistically similar.

### 3.3. Peripheral Blood T Cell Numbers were not Affected by the mTOR Inhibitor Conversion

**Figure 1:**
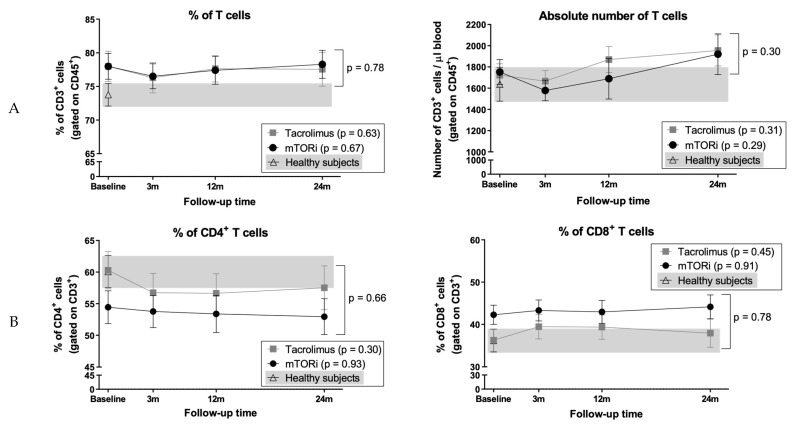
Evolution of T cells after switching from tacrolimus to serine/threonine protein kinase mechanistic target of rapamycin inhibitors (mTORi). Immunophenotyping of (**A**) total T cells and (**B**) CD4^+^ and CD8^+^ T cell subpopulations was carried out in patients before and after switching to mTORi (black dots) and in patients maintaining tacrolimus (grey squares). Healthy subjects (HS) data is depicted with white triangles, and the grey background corresponds to range. Plots show mean and standard error of the mean (SEM) for each time point.

Patients from both groups showed similar proportions and absolute numbers of circulating T cells during the study (Figure 1A). The proportions of T cells were greater in both groups at 24 months compared to HS (KT patients 78% ± 9.7% vs. HS 73.8% ± 7.6%, *p* = 0.035) (Figure 1A). Further analysis showed similar proportions of CD4^+^ and CD8^+^ T cells in both groups during the follow-up with no differences compared to HS (Figure 1B).

### 3.4. mTOR Inhibitors and Time Posttransplantation Promote Expansion of T Regulatory Cells

**Figure 2:**
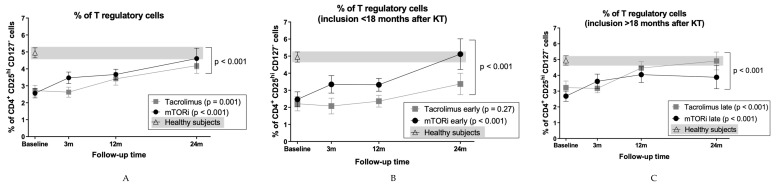
Evolution of Tregs after switching from tacrolimus to mTORi in all cases and according to time of inclusion in the study. Immunophenotyping of (**A**) total Tregs, (**B**) Tregs in patients included in the study during the first 18 months after transplantation, and (**C**) Tregs in patients included in the study after 18 months posttransplant. Patients before and after switching to mTORi are depicted with black dots, and patients maintaining tacrolimus are depicted with grey squares. HS data is depicted with white triangles, and the grey background corresponds to range. Plots show mean and SEM for each time point.

During follow-up, both groups showed a significant increase in the relative and absolute numbers of Tregs (mTORi baseline: 2.6% ± 1.5% and 49 ± 44 cells/µl, 24 months: 5.9% ± 4.3% and 111 ± 93 cells/µl, *p* < 0.001 for both; Tacrolimus baseline: 2.7% ± 1.3% and 47 ± 23 cells/µl, 24 months: 4.8% ± 2.4% and 95 ± 65 cells/µl, *p* = 0.001 and *p* = 0.01) (Figure 2A). The increase was lesser and delayed in the tacrolimus group (*p* < 0.001). A multivariable analysis assessing typical confounding variables such as recipient sex, age, delayed graft function, and time post-transplantation was performed. Results confirmed a significant influence of posttransplant time (*p* = 0.039) and sex (*p* = 0.050) but no influence of recipient age (*p* = 0.28) and delayed graft function (*p* = 0.90) in the increase of Treg cells (Table 2). As median time post-KT for inclusion was 18 months, we performed a sub-analysis considering patients enrolled “early” (<18 months) and “late” (>18 months) after KT (Figure 2B,C). Patients switched early to mTORi showed a significant increase in Tregs during the study (baseline: 2.5% ± 1.8%, 24 months: 5.1% ± 3.2%, *p* < 0.001), in contrast with no significant changes in early tacrolimus patients (baseline: 2.2% ± 1.2%, 24 months: 3.4% ± 1.7%, *p* = 0.27). Evolution between groups was significantly different (*p* < 0.001): proportions of Tregs in the mTORi but not in the tacrolimus group at 24 months were comparable to those of HS (5.1% ± 3.2% vs. 4.9% ± 1.3% *p* = 0.50, 3.4% ± 1.7% vs. 4.9% ± 1.3% *p* = 0.016) (Figure 2B). Patients switched late to mTORi showed an increase during the 24-month follow-up similar to those maintained on tacrolimus (tacrolimus baseline: 3.2% ± 1.2%, 24 months: 4.9% ± 1.6%, *p* < 0.001; mTORi baseline: 2.7% ± 1.2%, 24 months: 3.9% ± 2.2%, *p* < 0.001) (Figure 2C). Absolute numbers behaved similarly, and both groups showed comparable percentages to those of HS at 24 months (mTORi *p* = 0.08 and tacrolimus *p* = 0.94).

### 3.5. B Cells and Naïve B Cells Decrease After Conversion to mTOR Inhibitors 

**Figure 3:**
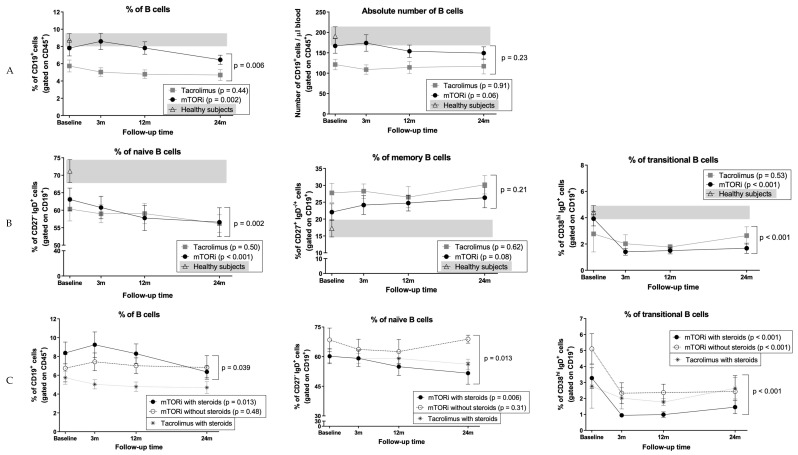
Evolution of B cells after switching from tacrolimus to mTORi. Immunophenotyping of (**A**) total B cells and (**B**) naïve, memory, and transitional B cells in patients before and after switching to mTORi (black dots) and patients maintaining tacrolimus (grey squares). HS data is depicted with white triangles, and the grey background corresponds to range. Plots show mean and SEM for each time point. Immunophenotyping analysis of (**C**) total, naïve, and transitional B cells in patients switching from tacrolimus to mTORi with (black dots) and without (white dots) concomitant steroid treatment. Patients who maintained tacrolimus and steroids are marked with (*) as a reference.

The proportions of B cells changed over time in the mTORi group, with a slight increase at 3 months followed by a continued decrease up to 24 months (baseline: 7.8% ± 4.9%, 24 months: 6.5% ± 2.5%, *p* = 0.002), whereas they remained stable in the tacrolimus group (*p* = 0.44), determining significant differences between groups (*p* = 0.006) (Figure 3A). No significant differences were observed in absolute numbers of B cells. Compared to HS, mTORi patients had similar proportions and numbers of B cells 3 months after conversion (8.6% ± 5% vs. 8.8% ± 3.3%) in contrast to tacrolimus patients (5.1% ± 2%), but at 24 months both groups depicted significantly lower percentages of B cells compared to HS (KT patients: 5.8% ± 2.6% vs. HS 8.8% ± 3.3%, *p* = 0.002) (Figure 3A).

Patients on mTORi showed a significant decrease of naïve B cells (baseline: 63.1% ± 17.5%; 24 months: 56.6% ± 19.4%, *p* < 0.001), but tacrolimus patients did not (*p* = 0.50), resulting in a different evolution during follow-up between groups (*p* = 0.002) (Figure 3B). Absolute numbers mirrored these changes (mTORi group *p* = 0.009, tacrolimus group *p* = 0.86, between groups *p* = 0.06). Memory B cells did not display significant changes (Figure 3B). Compared to HS, both groups of KT patients displayed lower percentages of naïve B cells (KT patients 56.4% ± 16% vs. HS 71.2% ± 14.6%, *p* = 0.001) and higher percentages of memory B cells at 24 months (KT patients 27.9% ± 12.8% vs. HS 17.3% ± 11.4%, *p* = 0.003) (Figure 3B).

### 3.6. Conversion to mTOR Inhibitors Promotes a Decrease in Circulating Transitional B Cells

We observed a significant decrease in the percentages of transitional B cells 3 months after mTORi conversion that persisted afterwards (baseline: 3.9% ± 2.9%, 24 months: 2.5% ± 3.2%, *p* < 0.001) compared to tacrolimus group (*p* = 0.53) (Figure 3B), confirmed by absolute numbers. Evolution of the two groups was different (*p* < 0.001), both in relative and absolute numbers (Figure 3B). Compared to HS, lower percentages and absolute numbers of transitional B cells in all KT patients were found at 24 months (KT patients: 2.1% ± 2.2% vs. HS 4.4% ± 2.2%, *p* < 0.001; 3 ± 3 cells vs. 8 ± 6 cells, *p* < 0.001) (Figure 3B). Further analysis of this subset revealed significant differences in the percentage of transitional B cell subset between both KTR groups at baseline (mTORi group 3.9% ± 2.9%, tacrolimus group 2.8% ± 5.5%, *p* = 0.004), probably mainly due to the absence of steroids in the treatment scheme of many patients in the mTORi group, as we revealed in a prior publication [43] (discussed next in Section 3.7).

### 3.7. Impact of mTOR Inhibitors on B Cells in Patients who did not Receive Steroids 

Our group recently showed that steroid withdrawal has a remarkable impact on B cell subpopulations [43]. Here, we stratified patients that changed to mTORi in two subgroups according to steroid treatment before starting the study (yes: *n* = 19; no: *n* = 10). The proportions of total B cells were significantly reduced at follow-up in patients on steroids (baseline: 8.4% ± 5.1%, 24 months: 6.4% ± 2.5%, *p* = 0.013) but not in steroid-free cases (*p* = 0.48); evolution between groups was different (*p* = 0.039) (Figure 3C). Steroid-free patients had higher percentages of naïve B cells at baseline and during follow-up in comparison with patients on steroids (*p* = 0.013), who showed significantly reduced naïve B cell proportions during follow-up (baseline: 60.2% ± 16.5%, 24 months: 51.6% ± 21.5%, *p* = 0.006) (Figure 3C); no differences in memory B cells were noticed. Finally, steroid-free patients displayed greater relative and absolute numbers of transitional B cells during follow-up compared to patients with steroids (between groups *p* < 0.001), though both subgroups showed a decrease in transitional B cells after conversion (both *p* < 0.001) (Figure 3C).

### 3.8. Conversion to mTOR Inhibitor Induces an Increase in the Proportion of CD56^bright^ Cells and NKG2A^+^ NK Cell Subsets 

**Figure 4:**
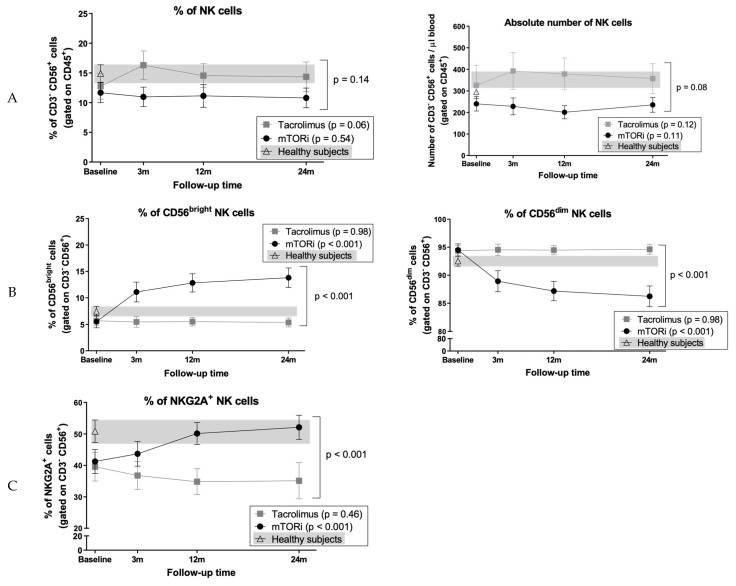
Evolution of Natural Killer (NK) cells after switching from tacrolimus to mTORi. Immunophenotyping of (**A**) total NK cells, (**B**) CD56^bright^ and CD56^dim^ NK cell subsets, and (**C**) NK cells expressing the CD94/NK group 2 member A (NKG2A) receptor. Patients before and after switching to mTORi are depicted with black dots, and patients maintaining tacrolimus are depicted with grey squares. HS data is depicted with white triangles, and the grey background corresponds to range. Plots show mean and SEM for each time point.

**Figure 5:**
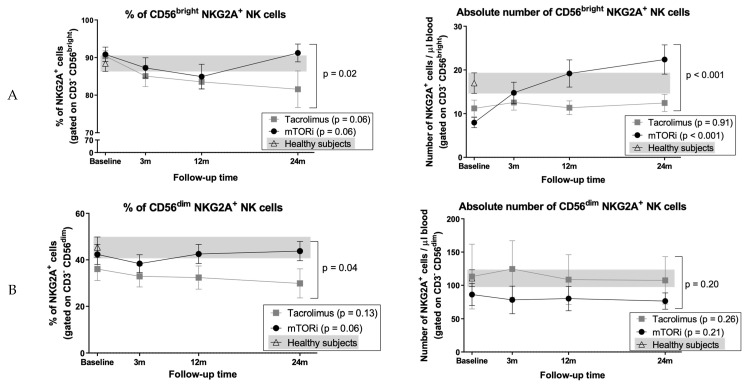
Evolution of CD94/NKG2A expression in NK cell subsets. Analysis of NKG2A expression was carried out gating (**A**) CD56^bright^ and (**B**) CD56^dim^ NK cells. Patients before and after switching to mTORi are depicted with black dots, and patients maintaining tacrolimus are depicted with grey squares. HS data is depicted with white triangles, and the grey background corresponds to range. Plots show mean and SEM for each time point.

Proportions and absolute numbers of total NK cells remained stable in both groups, and their evolution was comparable (Figure 4A). By contrast, proportions of CD56^bright^ NK cells increased in mTORi patients (baseline: 5.5% ± 5.7%; 24 months 13.8% ± 7.6%, *p* < 0.001) with a reciprocal reduction of the CD56^dim^ subset (baseline: 94.5% ± 5.5%; 24 months 86.2% ± 7.7%, *p* < 0.001) (Figure 4B). Conversely, NK cell subsets remained stable in the tacrolimus group (*p* = 0.98), resulting in significant differences between groups (both *p* < 0.001) (Figure 4B); calculation of absolute NK cell numbers confirmed these findings. A significant increase in the proportions of NKG2A^+^ NK cells occurred following mTORi conversion with levels similar to HS (baseline: 41.2% ± 20.6%; 24% months: 52.1% ± 18%, *p* < 0.001). By contrast, this parameter remained stable in the tacrolimus group (*p* = 0.46), accounting for differences between groups (*p* < 0.001) (Figure 4C); absolute numbers of NKG2A^+^ cells also increased in the mTORi group (*p* = 0.009; between groups *p* = 0.025). By contrast, no differences of NKG2C expression levels were observed. Since the majority of CD56^bright^ NK cells are NKG2A^+^, we compared the expression of this receptor in both CD56^dim^ and CD56^bright^ NK cell subsets. The numbers of CD56^bright^ NKG2A^+^ NK cells increased in the mTORi group (baseline 8 ± 6 cells/µl, 24 months 22 ± 14 cells/µl, *p* < 0.001), although the proportions varied during the study period (*p* = 0.06); and no differences were observed in the CD56^dim^ NKG2A^+^ NK cell subset (Figure 5A,B). Finally, considering previous results from our group [45], we analyzed these subsets excluding patients who developed HLA antibodies. No significant influence of HLA antibodies development was found.

## 4. Discussion

We report here that, after conversion to mTORi in KTR, graft function remained stable and there was no increase of dnDSA rate. However, there was a significant redistribution of PBL consisting of an increase in the proportion of peripheral regulatory T cells, CD56^bright^ and NKG2A^+^ NK cells, and a decrease in the proportion of transitional B cells.

Although conversion to mTORi has been associated with improvement in eGFR [46,47], we found no significant advantage in eGFR during two years of follow-up. Certainly, our study was not powered to assess graft function evolution. Proteinuria increased slightly and non-significantly in mTORi patients with no clinical impact, as earlier described [48].

Early conversion to mTORi without steroids may induce an under-immunosuppression state and an increased risk of dnDSA development [37]. These dnDSA are associated with the development of ABMR and worse graft survival [12]. In this study, we found no increased rates of dnDSA in late mTORi conversion for two years of follow-up, in agreement with several studies [40,41,49,50,51]. On the contrary, other studies have shown higher rates of dnDSA after conversion to everolimus [38,39,52]. Most data suggest that early (<1 year) mTORi monotherapy conversion may provide inadequate immunosuppression [36] and is a potential risk factor for dnDSA emergence, especially associated with steroid withdrawal [46,53]. In our study, only two cases developed dnDSA on mTORi, one converted early (3 months) after KT, and the other one not receiving steroids at the time of conversion.

Regarding the T cell immunophenotype, we found no influence of mTORi conversion on main T cell subsets, as previously reported in a study comparing de novo treatment with mTORi and cyclosporine A (CsA) after KT with a 2-year follow-up time [27]. In agreement with previous publications, we found a significant increase in regulatory T cells in the mTORi group [24,25]. Interestingly, patients recruited >18 months after KT and maintained on tacrolimus for the following two years also showed an increase of Tregs but not those included within the first 18 months after KT. Although multiple reasons can be linked to this phenomenon, lower dosage and trough levels of tacrolimus later after KT might explain the increase in Tregs. To our knowledge, this is the first study to show no difference in Treg expansion between tacrolimus and mTORi conversion >18 months after transplantation.

Analysis of the B cell compartment showed that only transitional B cells were affected by mTORi conversion, resulting in a striking reduction of their relative and absolute numbers only 3 months after conversion. The analysis of this B cell subset has gained interest during the last years as they potentially include regulatory B cells, which secrete interleukin-10 (IL-10) and show immunoregulatory properties [54,55]. Cherukuri et al. [56] suggested a clear implication of transitional B cells in renal allograft outcomes. In this study, transitional B cells were classified into T1 and T2 subsets (being T1 subset the one which expresses a higher ratio of IL-10 to tumor necrosis factor-alpha (TNF-alpha)) and they reported that a low T1/T2 ratio was independently associated with allograft dysfunction. On the other hand, regarding regulatory B cells, a study reported a similar reduction in mTORi and CNI patients [26] and another found no influence of sirolimus or tacrolimus in the proportion of these cells [23]. Regarding other B cell subsets, a study comparing the use of sirolimus and CsA de novo after KT reported an expansion of memory B cells and a reduction of naïve B cells [32]. In our study, other differences in B cell subsets, such as the reduction of total B cells and naïve B cells in mTORi patients, were related to the use of concomitant prednisone treatment. A previous study from our group showed that steroid withdrawal clearly modified B cell subsets, increasing the proportions of total, naïve, and transitional B cells [43]. In agreement with these results, we report here that steroid-free patients showed stable percentages of total B cells and naïve B cells after mTORi conversion whereas these cell populations decreased in steroid-treated patients. Naïve B cells are more sensitive to glucocorticoid-induced apoptosis [57,58], and mTORi may contribute to this overall effect. In contrast with CNIs, direct effects on B cells of mTORi have been reported [53]. In vitro cultures of human B cells with mTORi showed a profound attenuation of B-cell activation and IgG production [30,31]. Moreover, sirolimus but not tacrolimus was able to inhibit the proliferation of B cells and their differentiation into plasma cells [59].

Patients displayed a significant redistribution of NK cell subsets after switching to an mTORi, in agreement with reported data sustaining that the NK cell repertoire may be modulated by different immunosuppressive drugs [33]. Data from experimental studies in vitro and in mice [60,61] suggest different effects of sirolimus and everolimus in the inhibition of mTOR complex 1 (mTORC1) and mTOR complex 2 (mTORC2) that may be critical in terms of NK cell development and functionality and should be taken into account in human studies. Jin et al. [60] showed that everolimus was more efficient than sirolimus in inhibiting mTORC2 formation and mTORC2-dependent signaling in vitro. Data in mice suggest that mTORC1 sustains mTORC2 activity whereas mTORC2 acts as a repressor of mTORC1 to control NK cell effector functions [61]. In humans, the effects of mTORi in NK cells have been evaluated in vitro [33,34] and in vivo in combination with tacrolimus [33,34]. In agreement with a previous report [27], we did not find significant alterations in total NK cells. However, the expression of the CD94/NKG2A inhibitory receptor increased after mTORi conversion when compared to the tacrolimus group. Neudoerfl et al. [33] found that tacrolimus treatment was associated with slightly increased NKG2A expression in NK cells when compared to tacrolimus and sirolimus treatment, although it did not reach statistical significance. To our knowledge, the present study is the first to assess CD94/NKG2A expression in KTR after mTORi conversion without tacrolimus and to report a follow-up of two years. Interestingly, mTORi patients and HS displayed comparable proportions of NKG2A^+^ NK cells. We previously described an association of greater proportions of NKG2A^+^ cells with the presence of HLA DSA in KTR, both in cases treated with tacrolimus and in patients treated with mTORi without CNI compared to patients without HLA DSA [45]. In the present study, the low number of patients with HLA DSA precludes this analysis. We also observed a significant increase of CD56^bright^ NK cells, which produce cytokines but display low cytotoxic activity, and conventionally considered to represent an early maturation stage [62]. This minor subset lacks FcγR-IIIA (CD16), which triggers antibody-dependent cellular cytotoxicity (ADCC) and displays the CD94/NKG2A inhibitory receptor [34,62], which recognizes HLA-E [63], in the absence of inhibitory killer immunoglobulin-like receptors (KIR) specific for HLA class I molecules [64]. This phenotypic profile in mTORi patients was encompassed by a reciprocal reduction of the major CD56^dim^ NK cell subset, which mediates cytotoxicity and pro-inflammatory cytokine secretion [34]. Moreover, a significant increase of the proportions of NKG2A^+^ CD56^dim^ NK cells, conventionally considered to differentiate from the CD56^bright^ subset, was also detected. Altogether, these data support that conversion to mTORi therapy promotes a redistribution of the NK cell compartment characterized by the presence of greater numbers of NK cell subsets at early differentiation stages. Implications of this phenomenon remain unclear and deserve further assessment in prospective studies.

Our study has some limitations: First, the restricted number of patients included in the study who conversely underwent an extensive follow-up for two years. This fact, in addition to the high number of statistical tests performed in the results section, implies a weakness in terms of taking conclusions of individual results and increases the chance of type 1 error. Second, the absence of protocol graft biopsies precluding the analyses of potential correlations between peripheral lymphocytes and intragraft infiltrations. Finally, the absence of additional markers in the immunophenotypic analysis (e.g., CD16 and Foxp3), which would have allowed to more precisely characterize the influence of mTORi conversion on the lymphocyte subsets redistribution.

The strength of the present study relies on the long follow-up of KT recipients which permits to confirm that some of the changes observed in peripheral blood lymphocyte subsets after switching to an mTORi remain stable for two years. To our knowledge, this study is the first to evaluate significant immune markers such as NKG2A—previously linked to DSA detection [45]—in a cohort of KT patients treated with mTORi without concomitant tacrolimus, assessing in addition the influence of steroid treatment in peripheral blood lymphocyte subsets of KT recipients.

In summary, our study supports that mTORi conversion is safe in terms of renal function and dnDSA generation and influences the distribution of peripheral blood lymphocyte subsets, alone or in combination with steroid withdrawal. More studies analyzing the function and relevance of transitional B lymphocytes and NKG2A^+^ NK cells are necessary in order to better understand the immune and clinical implications of immunosuppressive treatment with mTORi.

## Figures and Tables

**Table 1:** Baseline and clinical follow-up characteristics of included patients: The table summarizes baseline characteristics and the clinical follow-up in patients switching from tacrolimus to serine/threonine protein kinase mechanistic target of rapamycin inhibitors (mTORi group) and patients maintaining tacrolimus (Tacrolimus group).

	Tacrolimus Group (*n* = 16)	mTORi Group (*n* = 29)	*p*-Value
Recipient age (years) (mean (SD))	52.4 (13.9)	52.5 (15.9)	0.990
Recipient sex (female) (*n*, %)	3 (19%)	9 (31%)	0.491
Race (caucasian) (*n*, %)	14 (88%)	25 (86%)	1.000
Type of donor (deceased) (*n*, %)	15 (94%)	25 (86%)	0.641
Donor age (years) (mean (SD))	46.9 (15.7)	43.2 (12.4)	0.385
HLA mismatch class I (A/B)/class II (DR) (mean (SD))	3 (1)/1 (1)	3 (1)/1 (1)	0.794/0.922
Sensitizing events before KT (yes) (*n*, %)	3 (19%)	9 (31%)	0.491
Induction immunosuppression (antilymphocyte antibodies) (*n*, %)	0 (0%)	2 (7%)	0.531
Delayed graft function (*n*, %)	4 (25%)	5 (17%)	0.700
Acute rejection pre inclusion (*n*, %)	1 (6%)	0	0.356
Anti-HLA DSA/no DSA antibodies prior to the study (*n*, %)	0 (0%)/0 (0%)	2 (6.9%)/1 (3%)	0.531/1.000
Time after KT (months) (median (p25-p75))	17.0 (3.0–48.8)	15.6 (3.3–50.1)	0.827
Immunosuppression treatment at inclusion			
CNI (*n*, %) MPA (*n*, %) Steroids (*n,* %)	16 (100%) 16 (100%) 16 (100%)	29 (100%) 23 (79%) 19 (66%)	
Immunosuppression treatment at the end of study *			
CNI (*n*, %) mTORi (*n*, %) MPA (*n*, %) Steroids (*n*, %)	16 (100%) 0 (0%) 16 (100%) 16 (100%)	6 (21%) 23 (82%) 21 (75%) 25 (89%)	
Renal function and proteinuria			
Creatinine at the start of study (mg/dL) (mean (SD))	1.4 (0.5)	1.3 (0.4)	0.286
eGFR at the start of study (mL/min/1.73 m^2^) (mean (SD))	57 (21)	59 (14)	0.763
pCOR < 500 mg/g at the start of study (yes) (*n,* %)	16 (100%)	29 (100%)	NA
Creatinine at the end of study (mg/dL) (mean (SD))	1.6 (0.8)	1.3 (0.5) **	0.246
eGFR at the end of study (mL/min/1.73 m^2^) (mean (SD))	56 (22)	61 (16) **	0.424
pCOR < 500 mg/g at the end of study (yes) (*n*, %)	16 (100%)	17 (77%) **	0.067

CNI: calcineurin inhibitor; pCOR: ratio protein/creatinine in urine; DSA: donor-specific antibodies; eGFR: estimated glomerular filtration rate; HLA: human leukocyte antigen; KT: kidney transplantation; MPA: mycophenolate acid; mTORi: mTOR inhibitor; NA: not applicable; SD: standard deviation * One patient associated tacrolimus to everolimus due to subclinical antibody-mediated rejection (ABMR). One patient died in everolimus treatment before the end of study and is not included in this count. ** From 22 patients at 24 months.

**Table 2:** Multivariable adjustment in the Generalized Estimating Equations (GEE) model for Treg cell numbers: The GEE population-averaged model analysis was also adjusted by time after KT, sex, age, and delayed graft function. Table shows the β and 95% confidence interval (CI) values and the *p*-value corresponding to each variable in the adjustment.

Adjusting Variable	β (95% CI)	*p*-value
Time Point of the Study	1.46 (0.57; 2.34)	0.001
Group of the Study Tacrolimus Group mTORi Group	0 1.32 (−19.63; 22.28)	0.901
Interaction Group and Time Point of the Study	0.10 (−1.04; 1.23)	0.868
Time After KT	2.97 (0.15; 5.80)	0.039
Recipient Sex Male Female	0 20.01 (0.01; 40.01)	0.050
Recipient Age	−0.35 (−0.99; 0.29)	0.281
Delayed Graft Function No Yes	0 1.62 (−24.13; 27.38)	0.902

CI: confidence interval; GEE: Generalized Estimating Equations; KT: kidney transplantation; mTORi: mTOR inhibitor.

## Supplementary Materials

The following are available online at https://www.mdpi.com/2077-0383/9/4/1088/s1. Figure S1: Gating strategy for flow cytometry analysis of PBL populations: Representative flow cytometry plots from the same patient illustrate the gating strategy used for the study; Figure S2: Characterization of transitional B cells; Table S1: Antibodies used in the study: The table summarizes antigen, clone, fluorochrome, and company for each antibody used in the study.
